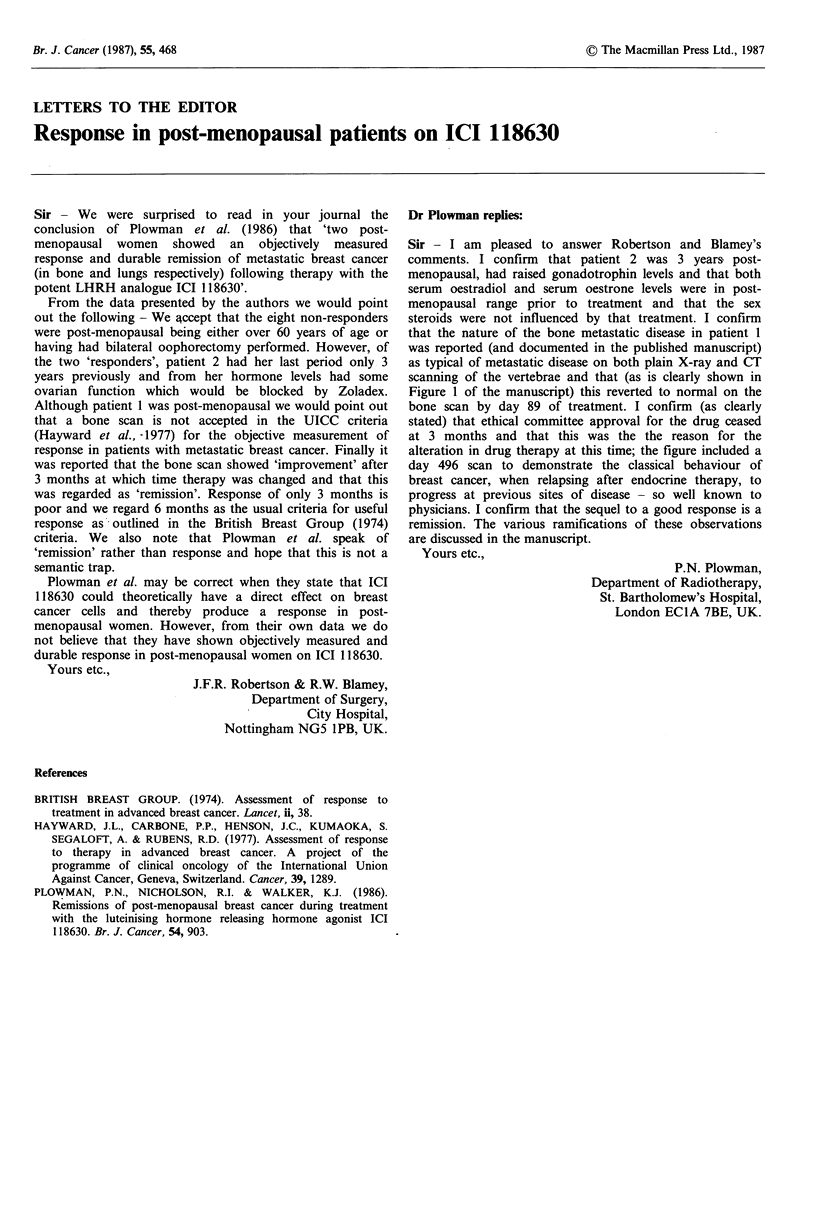# Response in post-menopausal patients on ICI 118630.

**DOI:** 10.1038/bjc.1987.93

**Published:** 1987-04

**Authors:** J. F. Robertson, R. W. Blamey


*Br. J. Cancer* (1987), **55**, 468 © The Macmillan Press Ltd., 1987

### LETTERS TO THE EDITOR

### Response in post-menopausal patients on ICI 118630

**Sir** – We were surprised to read in your journal the conclusion of Plowman *et al.* (1986) that 'two post-menopausal women showed an objectively measured response and durable remission of metastatic breast cancer (in bone and lungs respectively) following therapy with the potent LHRH analogue ICI 118630'.

From the data presented by the authors we would point out the following – We accept that the eight non-responders were post-menopausal being either over 60 years of age or having had bilateral oophorectomy performed. However, of the two 'responders', patient 2 had her last period only 3 years previously and from her hormone levels had some ovarian function which would be blocked by Zoladex. Although patient 1 was post-menopausal we would point out that a bone scan is not accepted in the UICC criteria (Hayward *et al.*, 1977) for the objective measurement of response in patients with metastatic breast cancer. Finally it was reported that the bone scan showed 'improvement' after 3 months at which time therapy was changed and that this was regarded as 'remission'. Response of only 3 months is poor and we regard 6 months as the usual criteria for useful response as outlined in the British Breast Group (1974) criteria. We also note that Plowman *et al.* speak of 'remission' rather than response and hope that this is not a semantic trap.

Plowman *et al.* may be correct when they state that ICI 118630 could theoretically have a direct effect on breast cancer cells and thereby produce a response in post-menopausal women. However, from their own data we do not believe that they have shown objectively measured and durable response in post-menopausal women on ICI 118630.

Yours etc.,

J.F.R. Robertson & R.W. Blamey,
Department of Surgery,
City Hospital,
Nottingham NG5 1PB, UK.

### References

BRITISH BREAST GROUP. (1974). Assessment of response to treatment in advanced breast cancer. *Lancet*, **ii**, 38.

HAYWARD, J.L., CARBONE, P.P., HENSON, J.C., KUMAOKA, S. SEGALOFT, A. & RUBENS, R.D. (1977). Assessment of response to therapy in advanced breast cancer. A project of the programme of clinical oncology of the International Union Against Cancer, Geneva, Switzerland. *Cancer*, **39**, 1289.

PLOWMAN, P.N., NICHOLSON, R.I. & WALKER, K.J. (1986). Remissions of post-menopausal breast cancer during treatment with the luteinising hormone releasing hormone agonist ICI 118630. *Br. J. Cancer*, **54**, 903.